# Aberration-free digital holographic phase imaging using the derivative-based principal component analysis

**DOI:** 10.1117/1.JBO.26.4.046501

**Published:** 2021-04-10

**Authors:** Xiaomin Lai, Sheng Xiao, Chen Xu, Shanhui Fan, Kaihua Wei

**Affiliations:** a Hangzhou Dianzi University, School of Automation and Artificial Intelligence, Hangzhou, China; b Boston University, Department of Electrical and Computer Engineering, Boston, Massachusetts, United States

**Keywords:** digital holographic microscopy, microscopy, phase imaging, aberration compensation, principal component analysis

## Abstract

**Significance:** Digital holographic microscopy is widely used to get the quantitative phase information of transparent cells.

**Aim:** However, the sample phase is superimposed with aberrations. To quantify the phase information, aberrations need to be fully compensated.

**Approach:** We propose a technique to obtain aberration-free phase imaging, using the derivative-based principal component analysis (dPCA).

**Results:** With dPCA, almost all aberrations can be extracted and compensated without requirements on background segmentation, making it efficient and convenient.

**Conclusions:** It solves the problem that the conventional principal component analysis (PCA) algorithm cannot compensate the common but intricate higher order cross-term aberrations, such as astigmatism and coma. Moreover, the dPCA strategy proposed here is not only suitable for aberration compensation but also applicable for other cases where there exist cross-terms that cannot be analyzed with the PCA algorithm.

## 1 Introduction

As we know, most natural cells are transparent in intensity images. Thus, stains or fluorescent dyes have been used to help visualize them. But, for some cases, such as living cultured cells, it is different to stain them. Moreover, long-term observation on fluorescent sample will cause photobleaching and phototoxicity. Phase imaging can ease these problems. For example, with the differential interference contrast microscopy,^1^ label-free living cells can be easily monitored. Nevertheless, this kind of method only records qualitative phase information. Digital holographic microscopy (DHM) can record quantitative phase information,^2^^,^^3^ with high sensitivity, accuracy, and resolution, in a noninvasive way. But, in this method, the sample phase is often embedded within aberrations, highly limiting its applications. Fortunately, these aberrations can be quantified and then compensated with physical or numerical methods.^4^^–^^8^

Physical methods are efficient, but not convenient, as they usually have strict requirements on an optical system, such as long-term stability. Compared with physical methods, numerical methods are more convenient and flexible. Thus, a lot of methods have been developed to quantify aberrations numerically, such as these using the 1D standard polynomials fitting,^9^^–^^12^ 2D least-squares surface fitting,^13^^,^^14^ Zernike polynomials fitting,^15^^,^^16^ and so on.^17^^–^^20^ However, a lot of numerical methods are only capable of compensating some specific aberrations,^14^^,^^19^^–^^22^ for example, only the lower order aberrations or non-cross term aberrations.

Another problem in numerical methods is the difficulty of separating the aberration phase from the sample phase. Some methods ignored the negative effect of sample phase,^13^ whereas others needed to detect the sample-free background regions.^5^^,^^9^^,^^15^^,^^16^ A recently developed numerical approach can automatically distinguish and compensate aberrations using the principal component analysis (PCA).^21^^–^^23^ In PCA, the first principal component (PC1) accounts for the largest possible variance in the dataset, with which aberrations can be automatically separated from the sample phase with much less perturbation.^21^^–^^23^ It is one of the most promising approaches. However, similar as other methods, a drawback of the PCA-based method is its inability of compensating cross-term aberrations.^5^^,^^23^ They need to use the 2D least-squares fitting as an additional step, to compensate the residual cross-term aberrations.^23^ The advantage of PCA algorithm was not fully preserved. Moreover, the unwanted sample phase appeared in the 2D least-squares fitting.^23^

The PCA algorithm cannot separate the cross-term aberrations because their singular values do not dominate in single component.^23^ Two or even more components must be used to recover the aberrations. But it is impractical because more components will also introduce sample phase. Thus, researchers thought that it was impossible to use the PCA-based methods to compensate for cross-term aberrations.^23^ Making their singular values dominate in single component is crucial. We discover that this can be realized by applying the PCA on the derivative phase instead of the original phase. Based on this idea, in this work, we propose a technique to provide aberration-free quantitative phase imaging in DHM, using a derivative-based principal component analysis (dPCA). The main hallmark of the presented dPCA approach is the compensation of all aberration, including the higher order cross-term aberrations. We show that with this method, the sample phase can be obtained without the disturbance of aberrations.

## 2 Methods

### 2.1 Principles

**Fig. 1:**
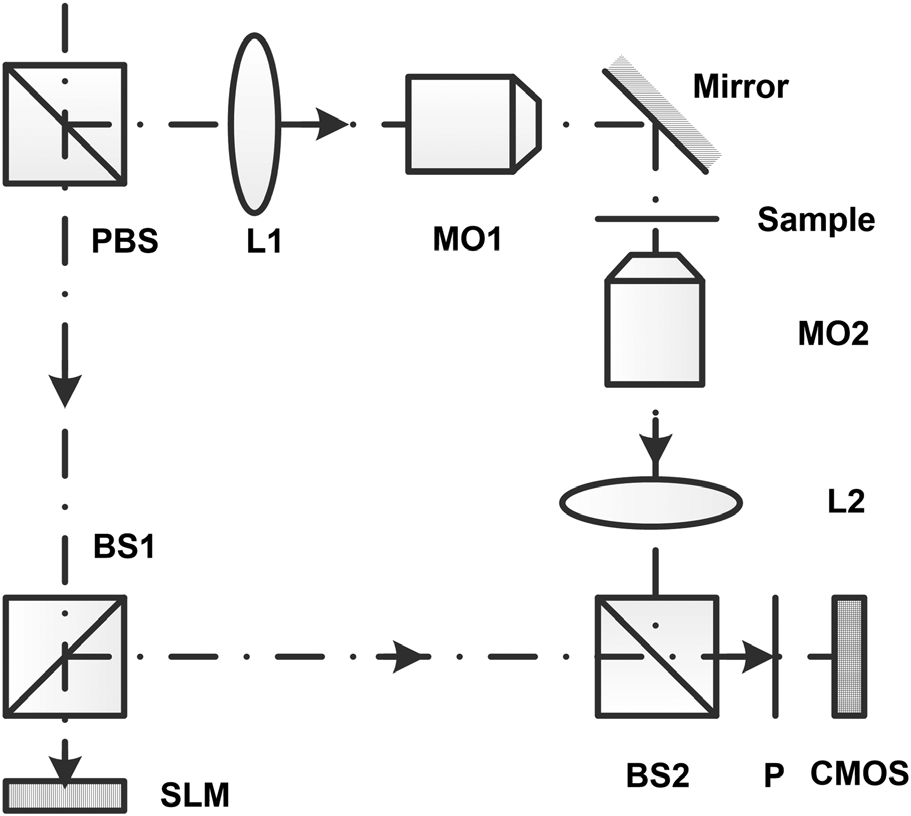
Configuration of DHM system. PBS, polarized beam splitter; BS, beam splitter; SLM, spatial light modulator; P, polarizer; L, lens; MO, microscope objective; CMOS, complementary metal oxide semiconductor.

This study is based on the experimental DHM system, presented in detail in Ref. 9. Figure 1 shows the configuration of the DHM system. Laser light of the central wavelength of 473 nm had been expanded and split into two beams with a polarized beam splitter (PBS). One beam passed beam splitter (BS) 1, spatial light modulator (SLM, Holoeye, Pluto-VIS), and BS 2 to act as a reference wave. The other beam was first collimated by the combining of lens L1 and microscope objective (MO) 1 to realized wide-field illumination. The transmitted light was collected by MO2 (Olympus, MPLFLN 20X/0.45) and L2 to act as an object wave. The reference wave and object wave were combined by BS2. A polarizer (P) before the detector (Thorlabs, DCC3260M) was adopted to adjust the intensity ratio between two beams. The SLM was used for phase shifting and introducing aberrations on purpose in some experiments.

It is well known that the intensity of hologram can be presented as

$$I=|O{|}^{2}+|R{|}^{2}+O^{*}R+OR^{*},$$ (1)

where O and R are the object and reference waves, respectively, and ${\left(\cdot \right)}^{*}$ denotes the complex conjugation.

By filtering the spectrum of off-axis hologram or using the phase-shifting holograms, the third or the fourth term in Eq. (1) can be separated, and then be used to obtain the object phase. Here, we use the fourth term, which is

$$OR^{*}=|O||R|\exp[i(\varphi_{o}+\varphi_{a})],$$ (2)

where $\varphi_{o}$ and $\varphi_{a}$ are the sample (object) phase and aberration phase, respectively. And $\varphi =\varphi_{o}+\varphi_{a}$ is the measured phase. To obtain the sample phase $\varphi_{o}$, aberrations $\varphi_{a}$ must be removed from Eq. (2).

Aberrations can be described with standard polynomials or Zernike polynomials. In this research, for convenience of presentation, they are described with the former one as

$$\varphi_{a}=\sum_{k=0}^{K}\sum_{l=0}^{L}P_{k,l}x^{k}y^{l},$$ (3)

where x, y are the coordinates, $P_{k,l}$ is the coefficient of aberration $x^{k}y^{l}$, k and l are the polynomial order of x and y, respectively. Parameters K and L represent the largest value of k and l, respectively. In Eq. (3), there are two types of aberrations. One type is the non-cross term aberrations in which k=0 or l=0, such as x, $x^{2}$, $x^{3}$, and y. The other type is the cross-term aberrations in which neither k nor l is zero, such as xy, $x^{2}y$, and $xy^{2}$.

**Fig. 2:**
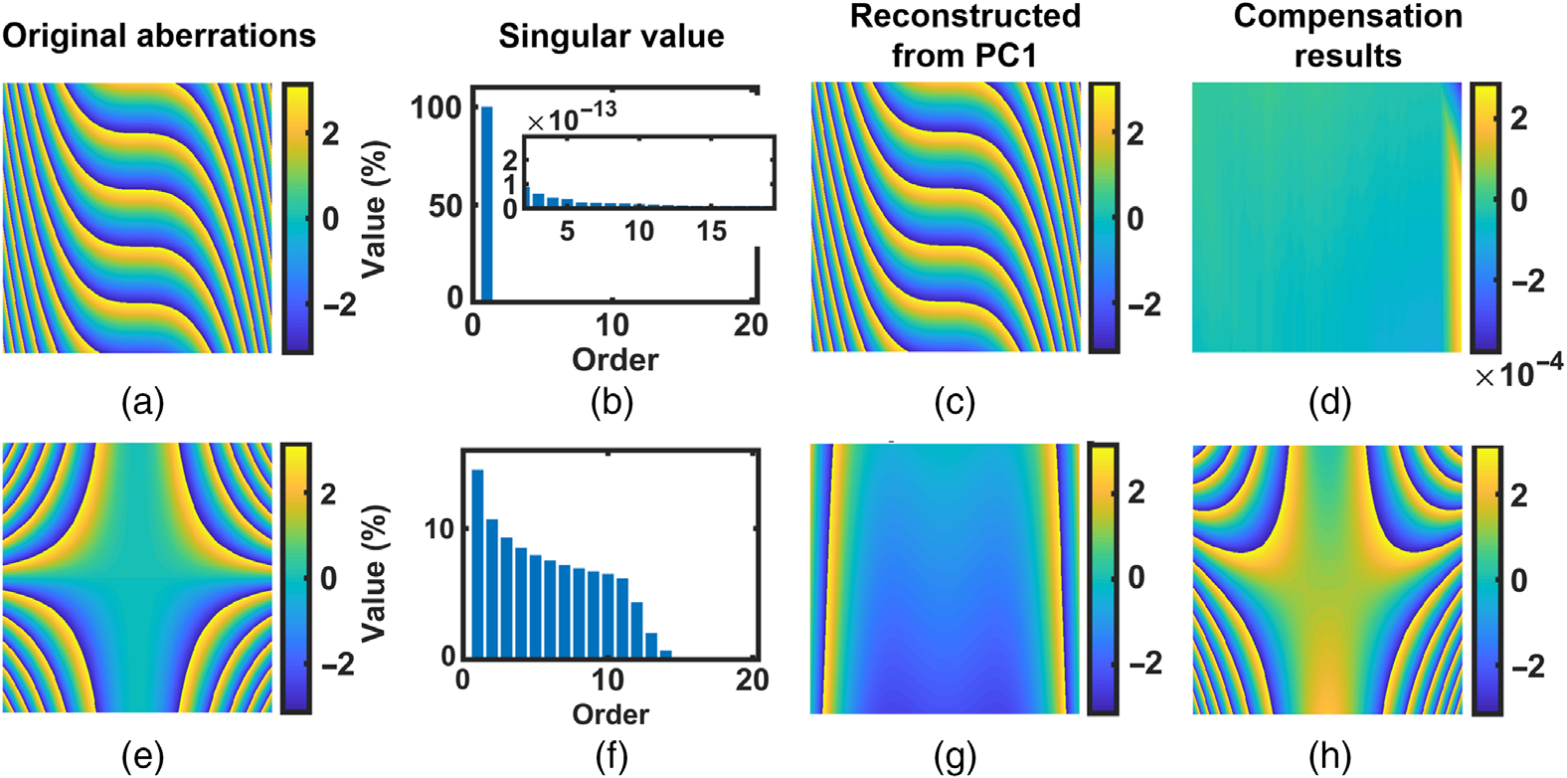
Traditional PCA compensation for (a)–(d) simulated non-cross term aberrations $P_{30}x^{3}+P_{01}y$ and (e)–(h) cross-term aberration $P_{21}x^{2}y$. For comparison, the inset shows the singular values of components 2 to 20. Color bars units: radian.

With PCA, the singular value of non-cross term aberrations will concentrate on the PC1, i.e., the principal component that has the largest singular value. Thus, these aberrations can be reconstructed from the PC1. The data in the first row of Fig. 2 confirm this point.

In the first row of Fig. 2, as an example, for the simulated non-cross term aberrations $P_{30}x^{3}+P_{01}y$ where $P_{30}=1\times 10^{-8}$ and $P_{01}=1\times 10^{-2}$ [Fig. 2(a)], we computed the percentage of variance (information) accounted for by each component and presented that of the first 20 principal components (PCs) in Fig. 2(b). In Fig. 2(b), we can see that the PC1 is dominant. The aberrations reconstructed from PC1 are shown in Fig. 2(c). By subtracting (c) from (a), we obtained the compensation result in (d). The aberration in compensation result is residual aberration. We can see that aberrations have been well compensated in Fig. 2(d) and the residual aberration is very small. Thus, as expected, the non-cross term aberrations can be compensated with the help of PCA algorithm.

However, the cross-term aberrations cannot be compensated simply with the PCA algorithm. Figures 2(e)–2(h) present the corresponding results for cross-term aberration $P_{21}x^{2}y$, where $P_{21}=1\times 10^{-8}$ [Fig. 2(e)]. In Fig. 2(f), the leading singular values were comparable instead of concentrating on a single one. Thus, in Fig. 2(g), the reconstructed phase using PC1 was largely deviated from the original one. Consequently, a large amount of residual aberrations existed after compensation [see Fig. 2(h)]. Therefore, the cross-term aberrations cannot be analyzed and compensated with the PCA algorithm.

As mentioned earlier, the PCA algorithm cannot reconstruct the cross-term aberrations because their singular values do not dominate in single component. Applying the PCA on the derivative phase instead of the original phase can solve this problem. That is because if we calculate their partial derivatives, the cross-term may be eliminated. To demonstrate this idea, we consider the general cross-term aberration as $f(x,y)=P_{k,l}x^{k}y^{l}$, where k≠0 and l≠0. Its “k’th order partial derivative with respect to x” and “l’th order partial derivative with respect to y” are

$$f_{x}^{k}=\frac{\partial^{k}f}{\partial x^{k}}=P_{k,l}k!y^{l},$$ (4)

$$f_{y}^{l}=\frac{\partial^{l}f}{\partial y^{l}}=P_{k,l}l!x^{k},$$ (5)

respectively, where the symbol ! denotes factorial function.

We can see that for cross-term aberrations, their partial derivatives (with proper order) could be non-cross terms while the coefficients $P_{k,l}$ are preserved. It indicates that the singular value of their partial derivatives could concentrate on single component. Thus, the partial derivatives can be reconstructed from the PC1. Then by adopting the reverse process of derivative (integral), the original cross-term aberrations before differential can be recovered, i.e., calculating the same order integral with respect to the same variable will recover the original aberrations.

**Fig. 3:**
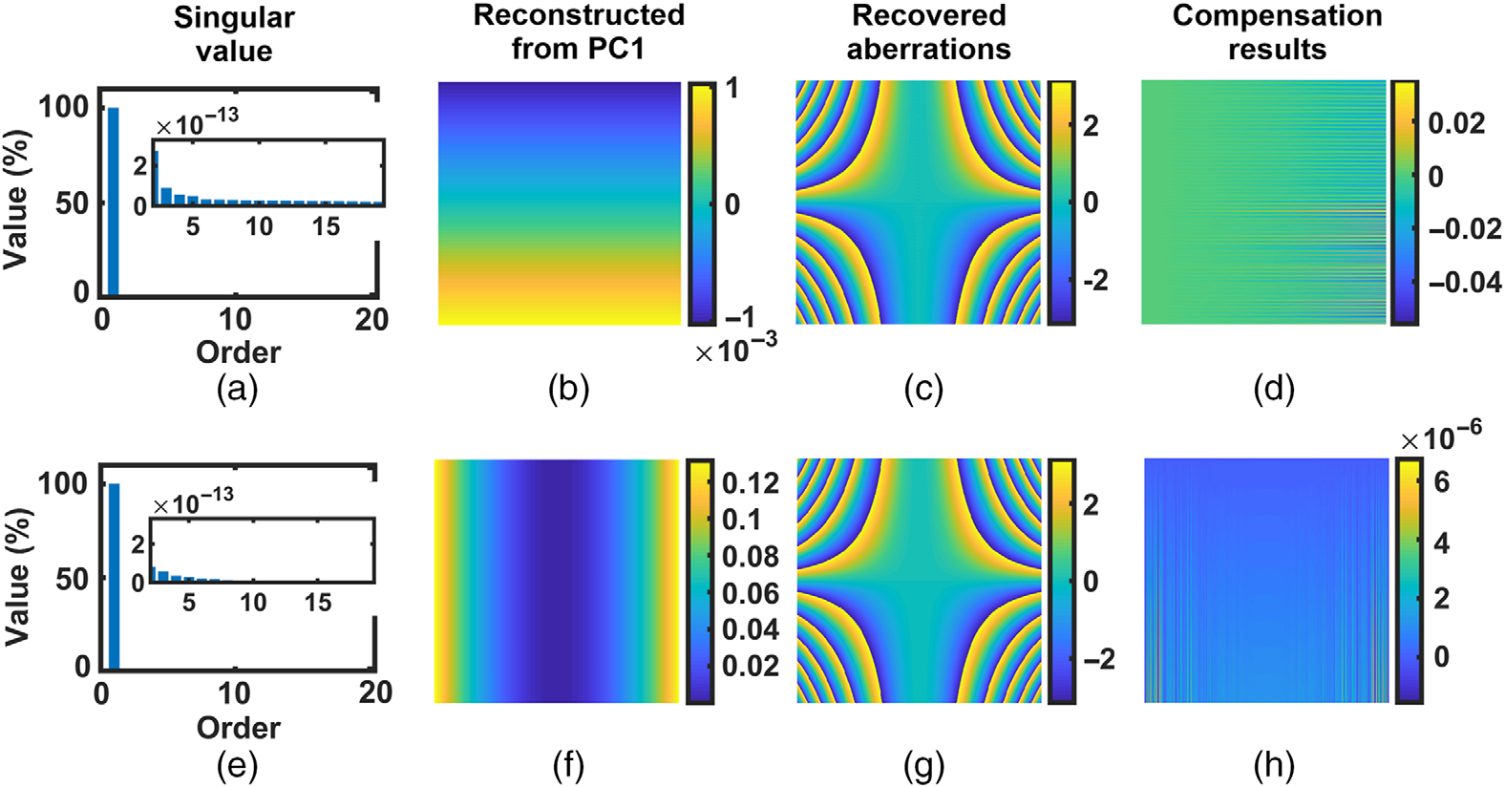
Derivative-based PCA compensation for simulated cross-term aberration $P_{21}x^{2}y$ [Fig. 2(e)]. The first row (a)–(d) presents the compensating results with the $2P_{21}y$, and the second row (e)–(h) presents that with the $P_{21}x^{2}$. The insets show the singular values of components 2 to 20. Color bars units: radian.

To demonstrate this concept, Fig. 3 presents the dPCA compensation process for the same cross-term aberration $P_{21}x^{2}y$ given in Fig. 2(e). In Fig. 3, the PCA was applied on its partial derivatives instead of itself. For aberration $P_{21}x^{2}y$, its “second-order partial derivative with respect to x” is $2P_{21}y$, whereas its “first-order partial derivative with respect to y” is $P_{21}x^{2}$. Both $2P_{21}y$ and $P_{21}x^{2}$ are non-cross terms. Thus, both of them can be analyzed using the PCA, as presented in the first and second row of Fig. 3, respectively.

In the first column of Fig. 3, as expected, the singular values of its partial derivatives concentrate on the PC1. Thus, the partial derivative of aberrations can be reconstructed from the PC1. The reconstructed partial derivative phases ($2P_{21}y$ and $P_{21}x^{2}$) are presented in the second column of Fig. 3. Then integrating over either x or y, we have the recovered aberrations, given in the third column of Fig. 3. The results after compensating with the recovered aberrations are shown in the fourth column. We can see that with the dPCA, cross-term aberration has been well compensated and the residual aberrations are very small, in agreement with the theoretical predictions. Therefore, cross-term aberrations can be compensated by applying the PCA on their partial derivatives. The dPCA strategy is capable of compensating the cross-term aberrations.

### 2.2 Compensation Procedure

**Fig. 4:**
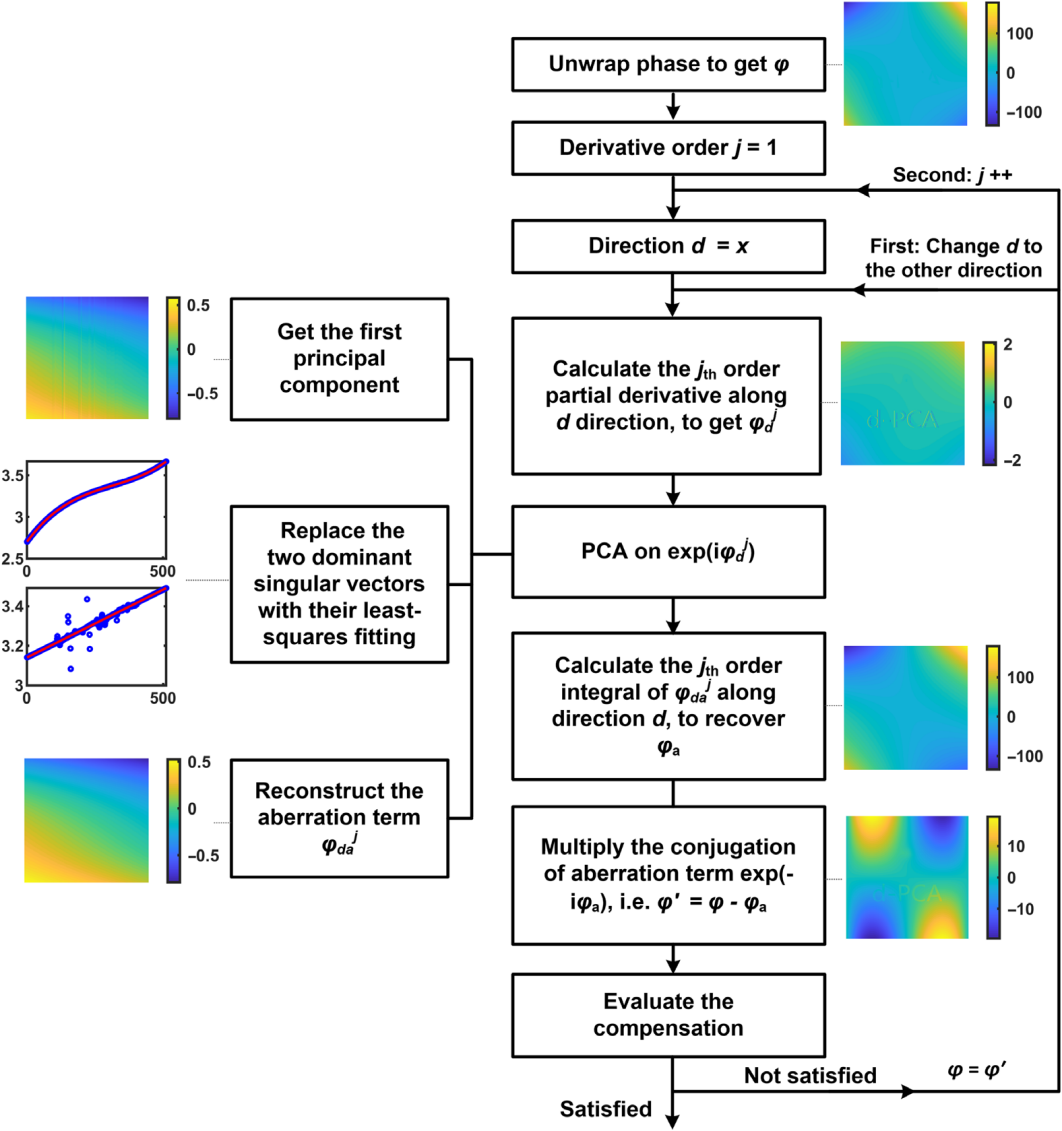
Flow diagram of the dPCA method. Color bars units: radian.

Based on these results, in Fig. 4, we present the overall procedure of compensating unknown aberrations with the dPCA strategy. First, the phase distribution is unwrapped^24^ and the derivative order j and direction d are set. We should point out that for the unknown phase distribution, which may be composed of many different orders of cross-terms, there is no need to determine the best order of derivative. We could simply start with order 1 and gradually increase it later. Thus, the initial derivative order j is set to 1. The initial direction can be either x or y. Usually, the dPCA should be implemented along both x and y directions. Which direction goes first does not matter. Here, we apply on x direction first.

Second, we compute the j’th order partial derivative with respect to d to obtain ${\varphi_{d}}^{j}$. The ${\varphi_{d}}^{j}$ are composed of sample phase information (${\varphi_{do}}^{j}$) and aberrations (${\varphi_{da}}^{j}$). Applying PCA on $\exp(i{\varphi_{d}}^{j})$, where $i^{2}=-1$, can reconstruct the aberration term (${\varphi_{da}}^{j}$), as presented in the left part of the flow diagram. The detailed process is that after extracting the PC1, the two dominant singular vectors of PC1 are replaced with their least-squares fittings, to get rid of noise. After this, use the PC1 to reconstruct the aberration term ${\varphi_{da}}^{j}$.

The reconstructed aberration ${\varphi_{da}}^{j}$ is not the aberration itself but its j’th order partial derivative with respect to d. We need to use the ${\varphi_{da}}^{j}$ to recover the real aberration $\varphi_{a}$. The aberration $\varphi_{a}$ can be recovered with the coefficients of polynomial fitting on ${\varphi_{da}}^{j}$ or with the inverse process of derivative, i.e., integral. Here, we use the later one. Thus, we set the unwrapped ${\varphi_{da}}^{j}$ as integrand and calculate its j’th order integral with respect to d to recover the real aberrations $\varphi_{a}$. Then, use the conjugation of $\varphi_{a}$ to compensate for aberrations as $\varphi^{\prime }=\varphi -\varphi_{a}$, where φ is the original phase, $\varphi_{a}$ is the recovered (or estimated) phase, and $\varphi^{\prime }$ is the compensation results. The aberrations in $\varphi^{\prime }$ are residual aberrations.

The next step is evaluating the performance of compensation either manually or automatically by calculating the standard deviation (STD) of some background regions.^9^^,^^12^ If aberrations have been well compensated, the compensation process is completed. Otherwise, update phase φ as $\varphi =\varphi^{\prime }$ and repeat the above process along the other direction. After this, if the residual aberration is still large, increase the derivative order and repeat the overall process until the residual aberration is small enough. We can see that the values of derivative order j and direction d are updated with iteration. For example, their values are j=1, d=x in the first iteration; j=1 and d=y in the second iteration; j=2, d=x in the third iteration; j=2, d=y in the fourth iteration, and so on. For normal optical system, aberrations are usually less than fourth order. Thus, fourth iterations are enough for normal optical system as they already cover up to fourth-order aberrations.

## 3 Results

**Fig. 5:**
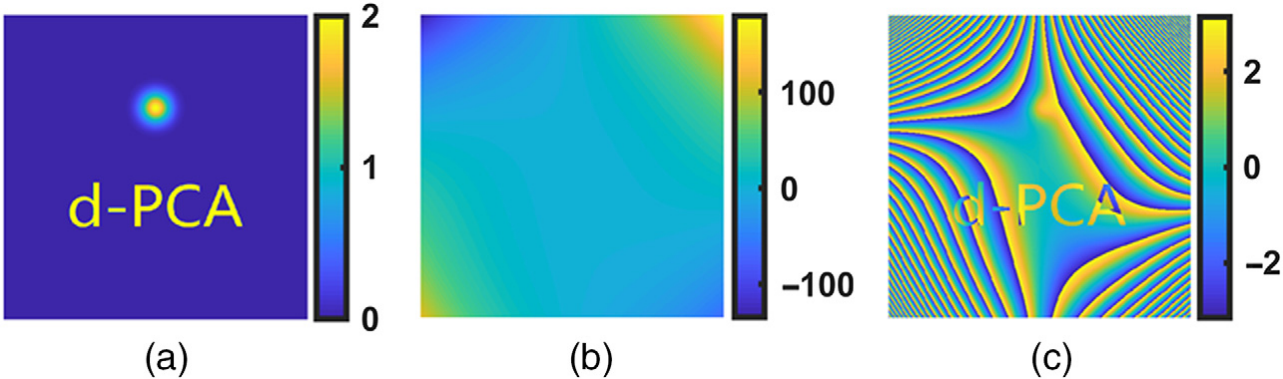
(a) Original object phase, (b) aberrations, (c) object phase disturbed by the aberrations in (b). Color bars units: radian.

To demonstrate this procedure, we present the process of obtaining the pure sample phase from a combined aberration, $\varphi_{a}=P_{20}x^{2}+P_{11}xy+P_{31}x^{3}y+P_{13}xy^{3}+P_{12}xy^{2}$, where $P_{20}=1\times 10^{-5}$, $P_{11}=1\times 10^{-5}$, $P_{31}=1\times 10^{-11}$, $P_{13}=1\times 10^{-11}$, and $P_{12}=1\times 10^{-8}$. The sample phase was consisted of gradually changed phase (a Gaussian spot) and sharply changed phase (letters “d-PCA”), showed in Fig. 5(a). The combined aberration $\varphi_{a}$ is presented in Fig. 5(b). Figure 5(c) is the object phase disturbed by the combined aberrations, presented in wrapped status.

**Fig. 6:**
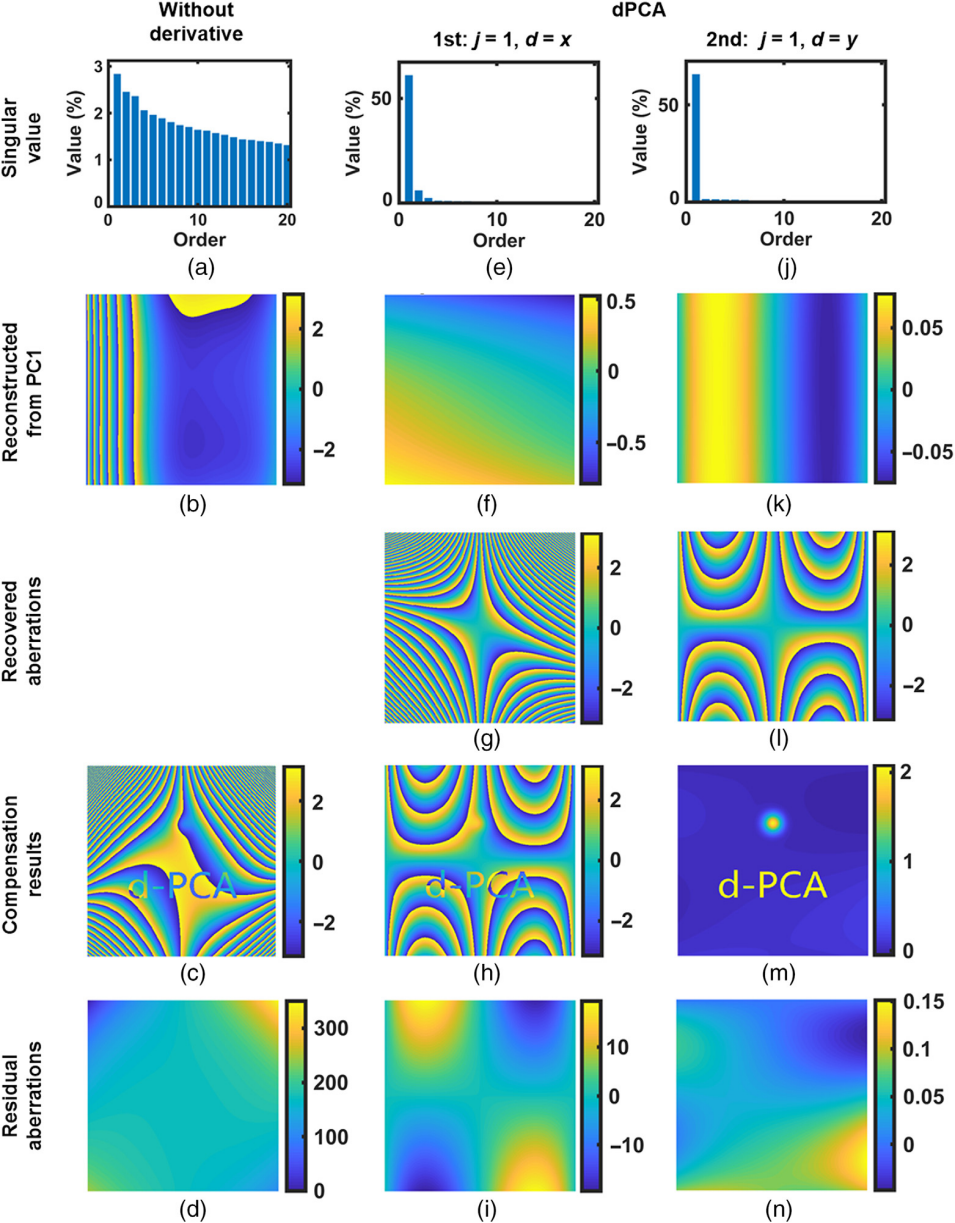
Compensation results for the unwrapped phase in Fig. 5(c), with the (a)–(d) traditional PCA and (e)–(n) dPCA. Results from the first and second iteration of the dPCA method are presented in the second and third column, respectively. Color bars units: radian.

For comparison, first, we used the traditional PCA (j=0) to compensate for aberrations. The results are given in Figs. 6(a)–6(d). The singular values of phase are given in Fig. 6(a), from which it is obvious that aberrations did not concentrate on a single component. Thus, in Fig. 6(b), the phase reconstructed from PC1 was different from the original ones. The compensation result in Fig. 6(c) was unacceptable as the object phase was still disturbed by aberrations. For clear observation, the difference between the original object phase [Fig. 5(a)] and the compensation result [Fig. 6(c)] is shown in Fig. 6(d), from which we can see that there are a lot of residual aberrations.

Figures 6(e)–6(n) present the compensation results using the dPCA method. The second column of Fig. 6 shows the results from the first iteration. In the first iteration, the PCA was implemented on the derivative phase ${\varphi_{x}}^{1}$, which was the “first-order partial derivative with respect to x” of the unwrapped phase in Fig. 5(c). In Fig. 6(e), the PC1 was dominated. The information of the data carried by the dominant PC1 was 9.7 times larger than that of the second principal component. Therefore, the aberration term (${\varphi_{xa}}^{1}$) in ${\varphi_{x}}^{1}$ can be reconstructed, as in Fig. 6(f). Calculating the first-order integral of ${\varphi_{xa}}^{1}$ with respect to x, we can recover the compensation phase $\varphi_{a}$, as given in Fig. 6(g). The compensation result using $\varphi_{a}$ is given in Fig. 6(h). The difference between the original object phase [Fig. 5(a)] and the compensation result [Fig. 6(h)] is shown in Fig. 6(i). There were residual aberrations in Fig. 6(i), because aberrations such as $P_{31}x^{3}y$ cannot be fully compensated with the ${\varphi_{x}}^{1}$. The compensation was not finished.

Thus, in the second iteration, we applied the dPCA on the other direction, i.e., the “first-order partial derivative with respect to y,” of the unwrapped phase in Fig. 6(h). The corresponding results are given in the third column [Figs. 6(j)–6(n)]. Figure 6(m) is the final compensation result. In Fig. 6(m), we can see that the object phase has been well revealed. The difference between the original object phase [Fig. 5(a)] and the final result [Fig. 6(m)] is shown in Fig. 6(n). The STD of residual aberration in Fig. 6(n) is 0.04 rad, indicating that aberrations have been well compensated with the dPCA method.

**Fig. 7:**
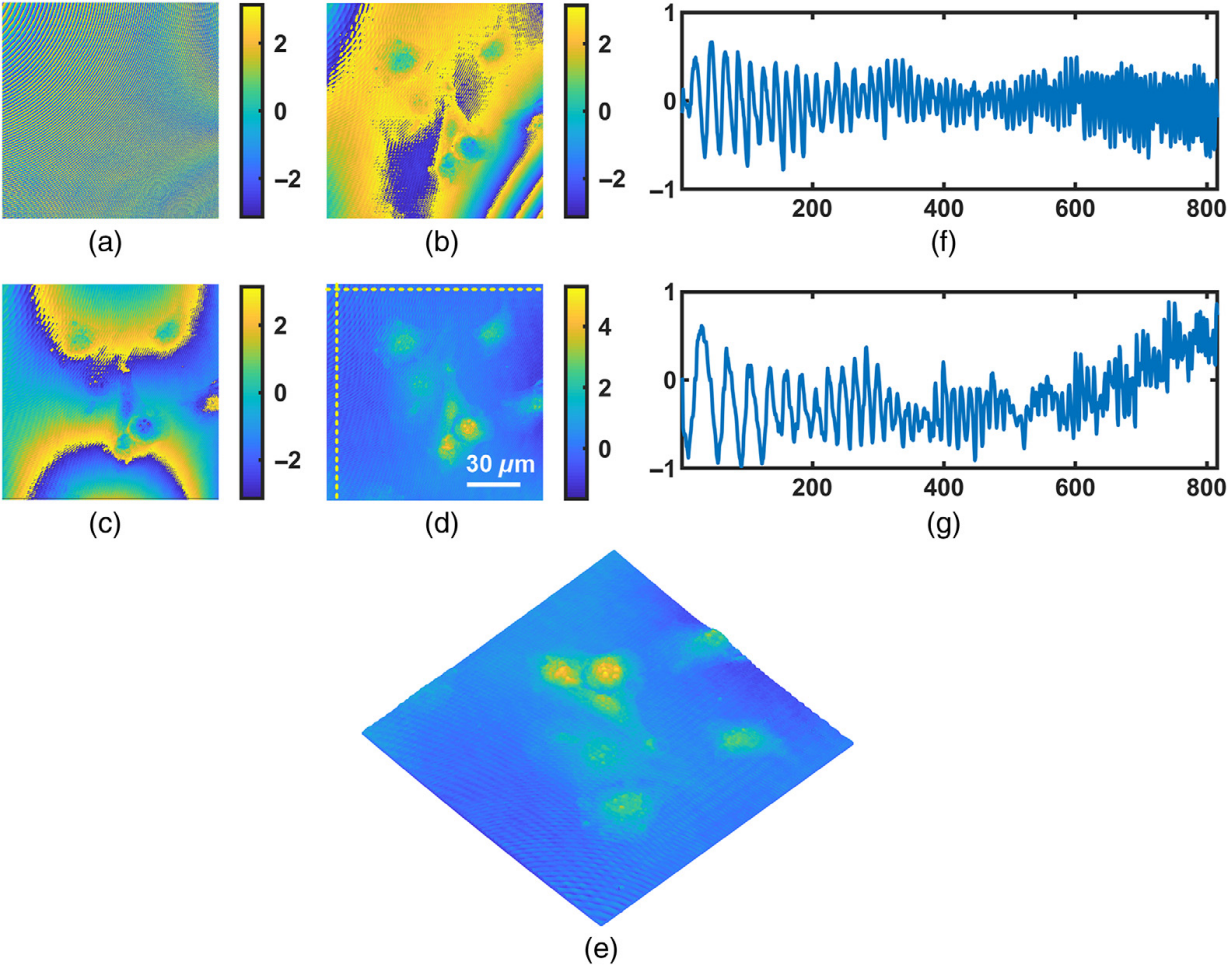
Compensation for aberrations in C2C12 cell phase image. (a) Original phase distribution, (b) compensation result with traditional PCA. (c) and (d) The results of the first and second iteration of the dPCA method, respectively. (e) The 3D view of (d). In (d) and (e), the phase value has been reversed for showing purpose. Profiles across the horizontal and vertical yellow lines of (d) are given in (f) and (g), respectively. Color bars units: radian.

In Fig. 7, we present the compensation results for the cell phase imaging. Figure 7(a) shows the murine myoblasts C2C12 cells phase alongside with aberrations. It is difficult to distinguish the cells in presence of a large amount of aberrations. Figure 7(b) shows the compensation result with traditional PCA, where the samples phase is still affected by residual cross-term aberrations. Figures 7(c) and 7(d) present the compensation results in the first and second iteration of the dPCA method, respectively. In the first iteration, the PCA was implemented on the first-order partial derivative of the unwrapped phase in Fig. 7(a) along the x direction, whereas in the second iteration it was implemented on the first-order partial derivative of the unwrapped phase in Fig. 7(c) along the y direction. The result after the second iteration is given in Fig. 7(d) in 2D view and in Fig. 7(e) in 3D view.

Profiles along the horizontal and vertical yellow lines of Fig. 7(d) are given in Figs. 7(f) and 7(g), respectively. The mean value of the profile in Fig. 7(f) is nearly flat, which means that the background has been compensated to flat. The STD of the profiles in Fig. 7(f) is 0.27. There is an increasement in Fig. 7(g) because it reaches the edge of another cell (not shown because it is out of field of view). We can see that aberrations have been well compensated. The morphologies of C2C12 cells can be clearly observed, including the nucleus. The object phase can be recognized without disturbance, again proving the effectiveness of this technique.

**Fig. 8:**
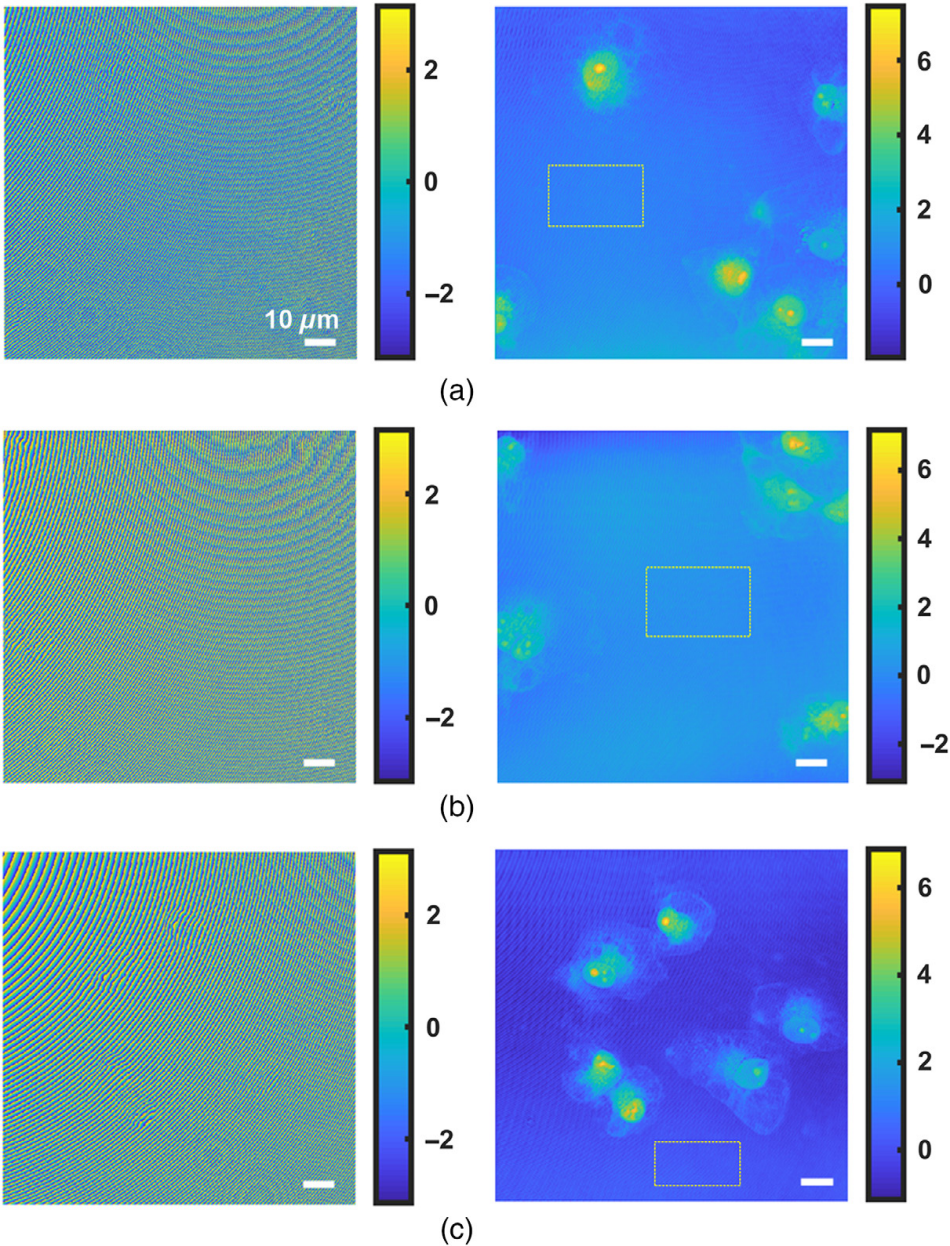
Compensation for aberrations in the phase image of MCF-7 cell. The locations of cells are more (a) random, (b) peripheral, and (c) concentrated. In each figure, the left and right column are the phase image before and after aberration compensation, respectively. Color bars units: radian.

We also tested the compensation results in the phase image of human breast cancer cells, MCF-7. The results are given in Fig. 8. In Fig. 8, the distributions of cells are different for each image, i.e., in (a) they are more random, whereas in (b) and (c) they are more peripheral and concentrated, respectively. For each figure, the left column is the phase image before aberration compensation while the right one is the phase image after compensation. The STDs of selected background regions (yellow squares) are (a) 0.25, (b) 0.15, and (c) 0.20, respectively. In Fig. 8, again we can see that aberrations have been well compensated and cells can be clearly observed after aberration compensation, proving the efficiency of dPCA method.

## 4 Discussion and Conclusion

We should point out that in this research, the derivative and integral were realized with MatLab functions “diff” and “cumsum,” respectively. In MatLab, the data in first several rows and columns were treated as initial values for differential and integral. These data are critical. Thus, we suggest that after the phase unwrapping, the data in first j (the highest order of differential) rows and columns should be replaced with their 1D least-square fittings to reduce the noise fluctuations. Meanwhile, before calculating the diff, the data in the first row and column should be saved, as they will be used as the initial data in cumsum.

Compared with conventional PCA (j=0) method, the dPCA method is more sensitive to noise, as it uses the derivative and integral. We suggest that one should combine the traditional PCA and dPCA method flexibly. For example, although the first-order partial derivative PCA also can compensate the non-cross term aberrations, we recommend trying the conventional PCA method first. If there are uncompensated cross-term aberrations, one could switch to the dPCA method and start with the first-order derivative PCA on the original aberrations data. Furthermore, if there are aberrations introduced by fitting errors when using the high-order derivative, an additional traditional PCA on compensation results can help remove them.

An advantage of the PCA-based method is that there is no need of background detection. The conclusion is based on the fact that the aberrations are dominated and can be separated with the PCA algorithm. If not, the sample phase will influence the compensation results. In this case, one could use methods such as the deep learning-based methods for background detection^5^ or direct compensation,^25^ if there are enough datasets and times to optimize the training network.

Moreover, in this work, we are mostly concentrating on the total phase aberration compensation, though at the cost of more computation time on 2D unwrapping, derivative, and integral. When using the MatLab installed in a 1.8 GHz laptop, the processing time on a 512×512 size image was 1.21±0.17  s for one iteration. We used the full-size PCA. One can use the spectrum-size-reduced PCA method^22^^,^^23^ to improve the computational efficiency if needed.

In summary, we have proposed a dPCA technique for aberration-free phase imaging. It solves the problem that the PCA algorithm was incapable of cross-term aberrations compensation and theoretically makes it possible to compensate for all aberrations. With the dPCA, aberration-free digital holographic phase imaging can be obtained, conveniently and efficiently, without requirements on background segmentation. It is an efficient total phase aberration compensation method. This technique will extend the application of the PCA algorithm and facilitate the biomedical application of DHM on quantitative phase imaging. Furthermore, the dPCA strategy proposed here is not only suitable for aberration compensation but also applicable for other cases where there exist cross-terms that cannot be analyzed with the PCA algorithm.

## Code, Data, and Materials Availability

The data that support the findings of this study are available from the corresponding author upon reasonable request.
